# High Thyroid Cancer Incidence Rate in a Community near a Landfill: A Descriptive Epidemiological Assessment

**DOI:** 10.3390/toxics9120325

**Published:** 2021-12-01

**Authors:** Maaike van Gerwen, Brandon Gold, Mathilda Alsen, Mohemmed N. Khan, Lauren Petrick, Eric Genden

**Affiliations:** 1 Department of Otolaryngology-Head and Neck Surgery, Icahn School of Medicine at Mount Sinai, New York, NY 10029, USA; brandon.gold@icahn.mssm.edu (B.G.); mathilda.alsen@mountsinai.org (M.A.); mohemmed.khan@mountsinai.org (M.N.K.); eric.genden@mountsinai.org (E.G.); 2 Institute for Translational Epidemiology, Icahn School of Medicine at Mount Sinai, New York, NY 10029, USA; 3 Department of Environmental Medicine and Public Health, Icahn School of Medicine at Mount Sinai, New York, NY 10029, USA; lauren.petrick@mssm.edu

**Keywords:** thyroid cancer, environmental exposures, screening, epidemiology

## Abstract

Background: to investigate the high thyroid cancer incidence rate of Staten Island and to disentangle the effects of potential environmental exposure from a landfill from screening. Methods: age-adjusted thyroid cancer incidence rates obtained from the New York State Public Access Cancer Epidemiology Data for New York State (NYS) excluding New York City (NYC) and the five NYC boroughs, including Staten Island, were mapped over time (1995–2018), investigated per age group and by percentage of localized thyroid cancer. Changes in trends were assessed using joinpoint. Contaminants of concern on Staten Island were assessed for carcinogenic and endocrine disruptive properties. Results: a more pronounced thyroid cancer incidence rate increase, without a difference in age distribution and similar percentages of localized thyroid cancer, was found in Staten Island compared to its demographic equivalent (NYS excluding NYC). Multiple contaminants of concern with carcinogenic and endocrine disrupting properties (e.g., cadmium, lead) were identified in air, water and sediment samples. Conclusion: investigations into the effects of increased/sustained environmental exposures are needed in chronically exposed populations to identify potential mechanisms of action of certain pollutants.

## 1. Introduction

Thyroid cancer incidence has been increasing since the 1970’s in the US [1]. Overdiagnosis of small, papillary thyroid cancers due to increased use and quality of diagnostic imaging explains only about 50% of this increased incidence, suggesting a true increase in the occurrence of thyroid cancer [2]. It has therefore been suggested that changes in the prevalence of environmental risk factors might play a role in thyroid cancer etiology and progression [2].

The thyroid cancer incidence rate of Staten Island, one of the five boroughs of New York City (NYC), is reported to be 67% higher compared to the rest of NYC [3]. The Fresh Kills Landfill, one of the world’s largest landfills and active between 1948 and 2001, is located on Staten Island. The landfill also contains World Trade Center (WTC) debris from the 9/11 disaster that has been linked to increased thyroid cancer in other studies [4,5,6]. The population of Staten Island continuously expressed concerns about potential adverse health effects associated with the landfill. In response to this, thyroid cancer screening sessions are being offered to residents of the borough at a regular interval from July 2017 [3]. From the literature, it is known that thyroid cancer screening is associated with diagnosis of smaller, lower stage cancers at a younger age [7].

There is currently limited understanding of the thyroid cancer etiology and most known or suspected risk factors include non-modifiable risk factors (e.g., increased age, female sex, non-Hispanic white, and positive family history) [2]. Modifiable risk factors considered to be associated with thyroid cancer include obesity and smoking [2]. On the other hand, certain environmental pollutants have also been reported to be associated with thyroid dysfunction and potentially thyroid cancer [8]. Exposure to ionizing radiation is a well-established modifiable environmental risk factor for thyroid cancer [2]; radioiodines (including Iodine-131 X-radiation, gamma radiation) are listed as thyroid cancer specific carcinogens [9]. Although increased population exposure to ionizing radiation through diagnostic exams may partially explain the worldwide increase in thyroid cancer, various endocrine disrupting chemicals (EDCs) (e.g., certain congeners of flame retardants, polychlorinated biphenyls (PCB), and phthalates, as well as certain pesticides) may also be linked to thyroid cancer [8,10]. Lastly, although exposure to metals has been suggested as a potential risk factor for thyroid cancer as higher thyroid cancer incidence rates have been reported in volcanic regions with elevated metal concentrations in soil, vegetation, and water, a recent meta-analysis showed that further research is needed to investigate the link with thyroid cancer for most metal ions [11,12,13].

Sources of pollution include urbanization and associated congestion, industrialization, waste facilities, and power plants. Contamination of air, water and soil has been implicated in the development of various other disease states, including infertility, cancer, metabolic and endocrine disorders, and asthma [8,14]. Serious health issues have been reported in populations living in close proximity to major toxic polluting sources. Residential proximity to heavy coal production in West Virginia was linked with poorer health status and increased risk of chronic lung disease, cardiopulmonary disease, hypertension, and kidney disease [15]. An ecological study in Spain, including residents near mining industries, suggested an association between proximity to the pollution source and excess mortality of colorectal cancer, lung cancer, bladder cancer and leukemia, as well as an association between underground coal instillation and thyroid cancer [16].

Geographical differences in the incidence of thyroid disease or cancer may additionally implicate a role for pollution as a risk factor. Excess risk of thyroid cancer was found in a number of National Priority Contaminated Sites in Italy. These sites are known to contain EDCs, which are exogenous substances or mixtures that interferes with endogenous hormone production and processing, due to the presence of major industrial and waste facilities [17]. EDC-polluted waters, resulting in contaminated fish products that are consumed by residents in coastal communities of Newfoundland, may have contributed to an elevated incidence of hypothyroidism in this population [18]. Clusters of thyroid cancer in the Hangzhou region in China were located in urban areas and regions with intensive industrial activities [19].

The higher thyroid cancer incidence rate on Staten Island, compared to the rest of NYC, offers a unique possibility to disentangle the roles of screening and environmental exposures. This is particularly important because excess diagnoses are associated with potentially unnecessary thyroid surgeries, the risk of surgical complications, as well as the necessity for life-long thyroid hormone replacement therapy [20]. This study, therefore, explored the potential roles of thyroid cancer screening and sustained environmental exposure associated with the Fresh Kills Landfill in the high thyroid cancer incidence rates of Staten Island, using a descriptive, epidemiological assessment.

## 2. Materials and Methods

### 2.1. Thyroid Cancer Data

Age-adjusted thyroid cancer incidence rates were obtained from the New York State Public Access Cancer Epidemiology Data (NYSPACED) for all New York State (NYS) counties and examined for the following NYS counties (NYC boroughs) separately: New York county (Manhattan), Kings county (Brooklyn), Queens county (Queens), Bronx county (Bronx), and Richmond county (Staten Island), and for the remaining counties combined (NYS excluding NYC) [21]. The NYS excluding NYC region most closely resembles the demographic composition of Staten Island [3]. In present paper, the NYC borough names are reported. In addition to NYS county/NYC borough information, year of diagnosis, age, and summary stage were extracted from the NYSPACED data files. Age-adjusted thyroid cancer rates by county were available from 1995 to 2018 in the used NYSPACED data release. Age was grouped into 19 age groups, starting with ≤1 year old and 1 to 4 years old, followed by 5-year age groups up to 80–84 years. All patients of 85 years and older were grouped into one age group. Summary stage was grouped into in situ, local, regional, distant, or unknown following the appropriate Surveillance, Epidemiology, ad End Results Program (SEER) staging variables.

To evaluate the thyroid cancer trend, age-adjusted thyroid cancer incidence rates of the five boroughs and NYS excluding NYC were mapped over time (1995–2018).

To assess changes in age-adjusted thyroid cancer incidence rates per geographic region, the method of joinpoint regression models was used to identify where different trend lines were connected at the “joinpoints” (Joinpoint Trend Analysis Software, version 4.9.0.0). The software starts with the minimum number of joinpoints and tests if more joinpoints are statistically significant, using a Monte Carlo Permutation method, and incorporating an estimated variation for each data point. The software also reports the Annual Percent Change (APC) for the trend lines. Because Staten Island most closely resembles the demographic composition of NYS excluding NYC, results of the joinpoint regression models of these two regions were reported. 

Age-adjusted thyroid cancer incidence rates per age group for the five boroughs and NYS excluding NYC were pooled for the years 1995–2000 and compared with the years 2010–2018 to evaluate the potential impact of thyroid cancer screening. The period 1995–2000 was chosen to minimize the effect of potential increased medical surveillance of responders who participated in rescue, recovery, and clean-up efforts at the WTC sites following the 9/11 disaster in 2001. The years 2010–2018 were combined because the increasing trend seemed to have stabilized.

The percentage of local/in situ stage thyroid cancer of Staten Island was compared to the percentages of the other four boroughs and NYS excluding NYC through multiple Chi-square tests using SAS software, version 9.4 (SAS Institute Inc., Cary, NC, USA).

### 2.2. Contamination Levels near the Fresh Kills Landfill

The Agency for Toxic Substances and Disease Registry (ATSDR) collected health data, environmental data, and community health concerns from the Environmental Protection Agency (EPA), health and environmental agencies, communities, and potentially responsible parties and published a Public Health Assessment regarding the Fresh Kills Landfill in 2000 at the request of concerned Staten Island residents [22]. This Public Health Assessment provides a review of the contamination levels near the Fresh Kills Landfill. The ATSDR determined chemical exposure pathways and routes of physical contact with the chemicals. To determine whether harmful levels of chemicals were present, the ATSDR screened the concentrations of contaminants against health-based comparison values (CVs), which were derived using very conservative assumptions. Contaminants of concern were defined as contaminants present at concentrations exceeding CVs [22].

The list of contaminants of concern published in the Public Health Assessment regarding the Fresh Kills Landfill was compared with the environmental risk factors for thyroid cancer identified by Fiore et al. [8,22]. For the risk factors present in both publications, their exposure pathway, source of contamination, route of exposure, time of chemical measurement, and concentration levels were retrieved from this Public Health Assessment. Furthermore, their carcinogenic hazards to humans was determined using the International Agency for Research on Cancer (IARC) Monographs [23]. The European Chemicals Agency (ECHA) classification was used to additionally identify carcinogenic/mutagenic hazards [24]. The overview of chemicals for tier 1 screening under the endocrine disruptor screening program published by the EPA in 2014 was used to assess potential endocrine properties of chemicals identified under the Safe Drinking Water Act (SDWA) [25]. Lastly, the Endocrine Society scientific statements were researched to assess additional endocrine disrupting properties of the identified contaminants of concern [26,27]. (The complete list of contaminants of concern is available as Supplementary Table S1).

## 3. Results

### 3.1. Age-Adjusted Thyroid Cancer Incidence Rates

**Figure 1:**
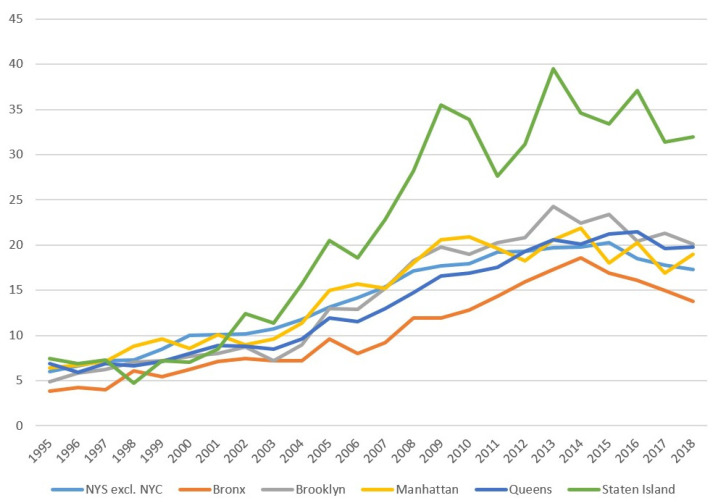
Age-adjusted thyroid cancer incidence rates for the NYC boroughs and NYS excluding NYC (1995–2018).

As shown in Figure 1, age-adjusted thyroid cancer incidence rates have increased substantially in all 5 five boroughs and NYS excluding NYC since 1995, however this increase was more pronounced on Staten Island since the early 2000’s.

**Figure 2:**
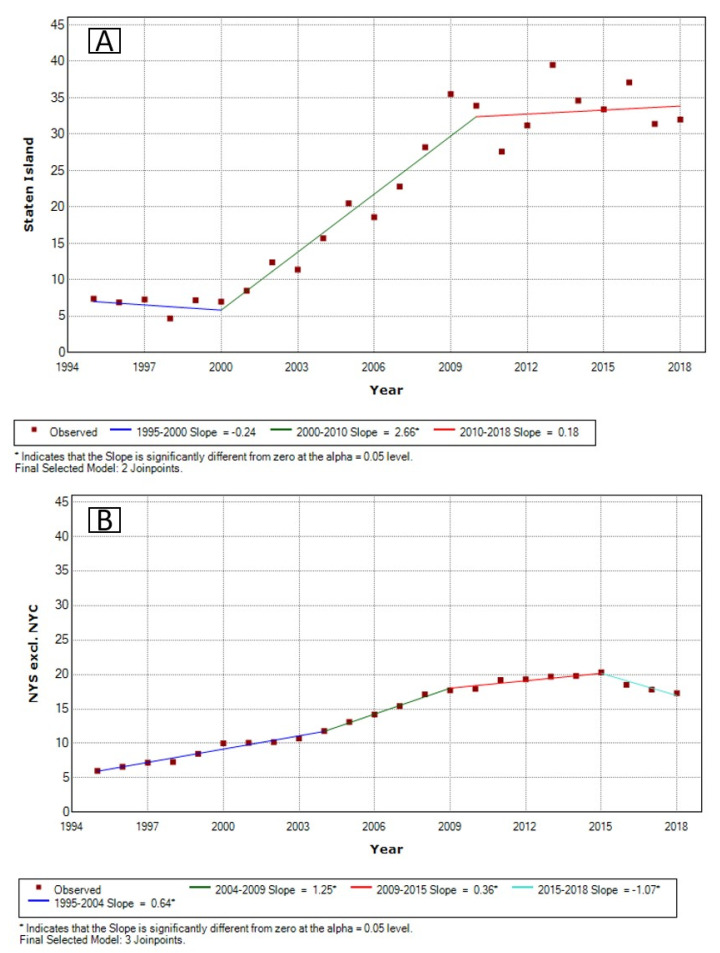
Annual percent change in age-adjusted thyroid cancer rates on Staten Island (**A**) and NYS excluding NYC (**B**) between 1995 and 2018.

Joinpoint analyses showed a significant increase in age-adjusted thyroid cancer rates on Staten Island (APC: +2.7% (*p* < 0.001)) between 2000 and 2010. Age-adjusted thyroid cancer rates in NYS excluding NYC increased significantly through 2015: 1995–2003: APC +0.6% (*p* < 0.001); 2004–2009: APC +1.3% (*p* < 0.001); 2009–2015: APC +0.4% (*p* = 0.033). On Staten Island, no significant up- or downward trend was found before 2000 (*p* = 0.670) or after 2010 (*p* = 0.727). From 2015, the age-adjusted thyroid cancer incidence rate in NYS excluding NYC significantly decreased (APC: −1.1% (*p* = 0.007)) (Figure 2).

**Figure 3:**
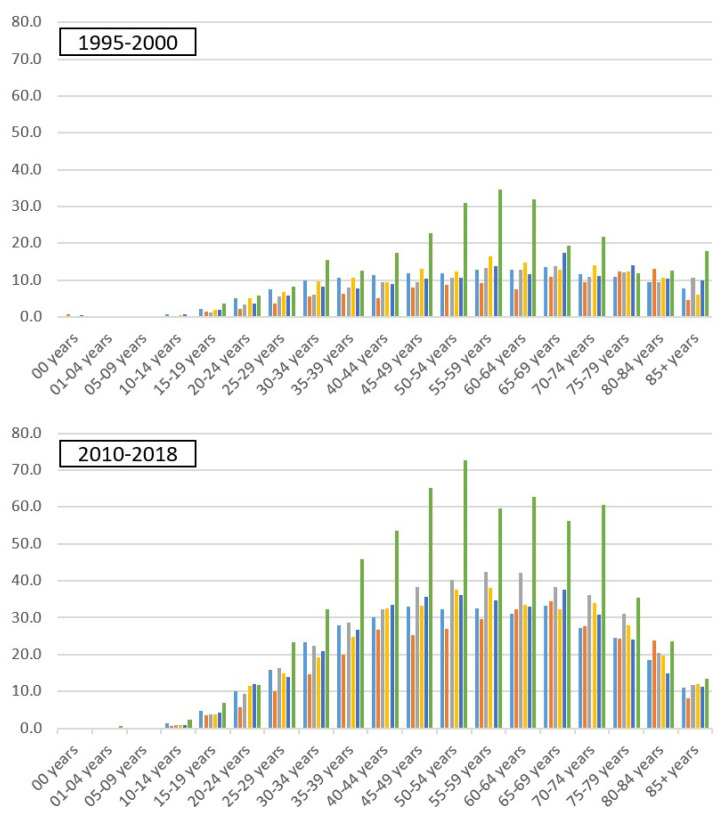
Age-adjusted thyroid cancer incidence rates per age group for the NYC boroughs and NYS excluding NYC for 1995–2000 and 2010–2018.

Figure 3 shows no pronounced difference in age distribution at thyroid cancer diagnosis between the periods 1995–2000 and 2010–2018 comparing Staten Island with the other four boroughs and NYS excluding NYC. Although Staten Island age-adjusted thyroid cancer incidence rates were high for various age groups in 2010–2018, especially for 45–49 years, 50–54 years, 55–59 years, and 60–64 years, there doesn’t seem to be a shift to higher thyroid cancer rates in younger age groups on Staten Island compared to the other geographic regions.

**Figure 4:**
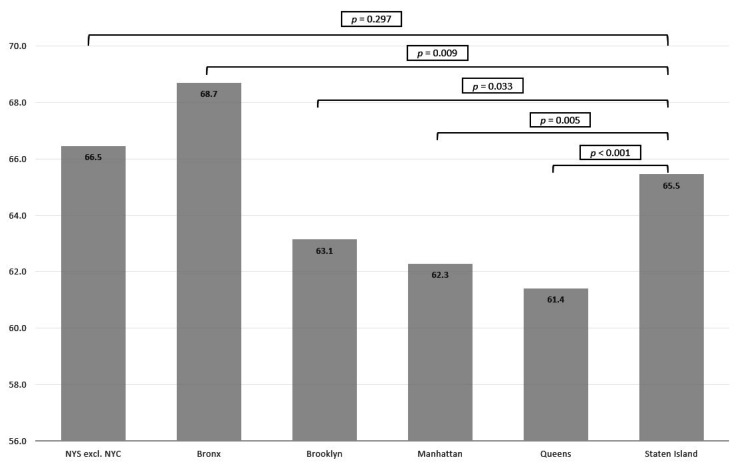
Percentage of local stage/in situ thyroid cancer per geographical region.

On Staten Island, 65.5% of the thyroid cancers were local stage/in situ cancers at diagnosis; the Bronx (68.7%) had a significantly higher percentage of local stage/in situ cancer compared to Staten Island (*p* < 0.001). There was no significant difference in percentage of local stage/in situ cancer when comparing Staten Island with NYS excluding NYC (*p* = 0.297). Brooklyn (63.1%), Manhattan (62.3%) and Queens (61.4%) had significantly lower percentages of local stage/in situ cancer compared to Staten Island (Figure 4).

### 3.2. Potential Exposures from the Fresh Kills Landfill

Particulate matter containing cadmium at peak concentrations exceeding CV was found in the air, potentially exposing Staten Island residents through inhalation (Table 1). Cadmium, as well as lead, was also found at levels exceeding CV in surface water, sediment and fish/shellfish). Vanadium was found at levels exceeding CV in surface water and sediment. Fish and shellfish additionally contained levels exceeding CV of dioxins, PCBs and DDT (Table 1).

Cadmium has been classified as a group 1 carcinogen and has endocrine disrupting properties. Lead and vanadium have been classified as group 2B carcinogens. Dioxins (group ≥ 1 carcinogen), PCBs (group 1 carcinogen) and DDT (group 2A carcinogen) all have endocrine disrupting properties (Table 1).

## 4. Discussion

A more pronounced increase in thyroid cancer incidence rate was seen on Staten Island compared to the other NYC boroughs and NYS excluding NYC, in particular after 2000. Although there might be some indications that environmental exposure may play a role besides screening (e.g., no shift towards thyroid cancer diagnosis at younger age, no significant difference in local stage/in situ cancer between Staten Island and NYS excluding NYC), current study does not allow for estimating the effect of screening. However, thyroid-health/cancer related environmental risk factors exceeding comparison values and with carcinogenic and endocrine disrupting properties have been identified in air, water, sediment and food chain samples collected on Staten Island between 1990 and 1999. Rigorous research investigating the thyroid cancer population of Staten Island population is therefore needed to assess the potential effects of sustained environmental exposure associated with the Fresh Kills Landfill.

Although thyroid cancer incidence has increased steadily in the US and worldwide over the past decades, a more dramatic increase has been found in countries with thyroid cancer screening [1,28]. A well-known example is the thyroid cancer “epidemic” in the Republic of Korea following the implementation of a government-initiated national screening program in 1999, which resulted in a 6-fold increase in thyroid cancer incidence between 1999 and 2008 [7]. As a result of thyroid cancer screening, the mean tumor size decreased significantly from 21.5 mm to 10.5 mm (*p* < 0.01) between 1999 and 2008, respectively [7]. Furthermore, a 7-fold increase of local stage tumors was found between 1999 and 2008 [7]. The mean age at diagnosis however slightly increased from 46.0 to 46.8 years (*p* = 0.03) between 1999 and 2008. The present study also found a more pronounced increase of thyroid cancer incidence on Staten Island compared to the other NYC boroughs and NYS, which may indicate an effect of the implementation of screening. However, age at diagnosis, as well as percentage of localized/in situ cancers, did not dramatically differ when compared to the other boroughs. The Bronx even had a significantly higher percentage of localized/in situ thyroid cancers compared to Staten Island. When comparing Staten Island to NYS excluding NYC, which more closely resembles the demographic composition of Staten Island, no difference was found in the percentage of localized/in situ cancers. An epidemiological assessment of the WTC first responders’ cohort, a group under greater medical surveillance with increased thyroid cancer incidence, showed that tumor size and age at diagnosis did not differ from unexposed thyroid cancer cases treated at Mount Sinai Hospital, suggesting that exposures may play a role [29]. The current study did not find a significant thyroid cancer incidence increase following the implementation of thyroid cancer screening on Staten Island in 2017, with the caveat that only data up to 2018 were available for analysis. It is also unlikely that intensified screening of WTC first responders following the 9/11 disaster in 2001 resulted in the increasing thyroid cancer incidence trends in Staten Island. Although exact data regarding the residence of WTC first responders is not publicly available, data from the 2011–2015 American Community survey showed that 2.6% of the workforce in Staten Island was engaged in firefighting/closely related professions, versus 2.9% in the Bronx [3]. Furthermore, as the fire fighter cohort consists of mainly males, it does not explain the increasing thyroid cancer incidence rates among both males and females on Staten Island (APC: +0.92% (*p* < 0.001) and APC: +2.61% (*p* < 0.001), respectively). (Supplementary Figures S1 and S2) Future research into exposure-related carcinogenic mechanisms potentially associated with pollutants from the Fresh Kills Landfill seems warranted to further investigate the Staten Island “phenomenon”, especially since multiple contaminants of concern with carcinogenic and endocrine disrupting properties were identified in investigated samples.

It is important to highlight cadmium, which was identified in air, surface water, sediment, and food chain samples. Cadmium is a heavy metal, which are known to have endocrine disruptive properties, and its carcinogenic mechanisms are multifold. Cadmium inhibits antioxidant enzymes and induces oxidative stress to activate the PI3K and ERK intracellular pathways, deregulate cell proliferation and damage DNA repair mechanisms [11]. It also favors tumor progression and invasiveness through the disruption of E-cadherin, thus destabilizing intercellular adhesion [11]. Cadmium also exhibits metalloestrogen properties that may contribute to a predilection towards thyroid cancer by replicating the effects of 17β-estradiol on the G protein-coupled estrogen receptor present on thyroid follicular cells. In turn, cadmium promotes proliferation, invasion, and migration of thyroid cancer cells [11]. Furthermore, cadmium accumulation in the thyroid has been well demonstrated. One study showed that the concentration of cadmium in thyroid tissue was three times higher in those living in cadmium-polluted areas versus people living in non-polluted areas [30]. Higher incidence of thyroid cancer has also been reported in volcanic regions, where water, soil and vegetation contain elevated levels of heavy metals, including cadmium [31]. Given the carcinogenic mechanisms, together with a long biological half-life of 10–30 years, cadmium seems to be a rather significant contaminant of concern for thyroid cancer outcomes [32].

Another important concern is that mixtures of various compounds may act additively or even synergistically to potentiate carcinogenic effects. A systematic review that analyzed the association between exposure to mixtures of persistent, bioaccumulative and toxic chemicals and cancer risk found that many of the mixtures examined in the literature are potentially more strongly associated than each individual contaminant with particular neoplasms [33]. To illustrate, a recent study found that exposure to a mixture of heavy metals is positively associated with cancer mortality, as well as all-cause mortality. In fact, this study implicated cadmium as having the highest contribution to this, which is highly relevant given cadmium was identified as a pollutant of concern on Staten Island [34]. Furthermore, a study in Italy highlighted that patients with head and neck cancer, including thyroid cancer, were more likely to have both elevated levels of heavy metal ions, such as cadmium and arsenic, and PCBs compared to their healthy counterparts [35]. The effects of these interactions between compounds need to be further explored to identify specific synergistic and antagonistic interactions. The presence of contaminant mixtures represents another potential factor associated with the high thyroid cancer burden on Staten Island.

It is important to note that the present study reported the results of a descriptive epidemiological assessment of thyroid cancer characteristics and trends combined with a summary of previously published contaminant assessments on Staten Island so no causal inferences can be derived, which is a limitation. Another limitation is that no detailed information is available on the exact screening and surveillance programs on Staten Island (e.g., targeted specific populations/age groups), therefore limiting the conclusion regarding the impact of screening on the thyroid cancer incidence rates to general remarks. Although the demographic composition of Staten Island most closely resembles NYS excluding NYC (e.g., smaller proportions of Asians, Hispanics, and foreign-born people of all races and ethnicities), it cannot be excluded that other important characteristics affecting the conclusions drawn from the local/in situ percentages regarding the impact of screening differ between these two regions [3].

## 5. Conclusions

The potential role of environmental pollution in health issues, including cancer, is of increasing concern due to the ubiquitous presence of multiple pollutants, chemicals and metals in the environment. The thyroid, because of its biology and biochemistry, may be sensitive to environmental pollution, however data on the carcinogenic effect of many environmental pollutants is currently lacking. Investigating thyroid cancer in an area with increased and sustained environmental exposures, such as Staten Island, provides the unique possibility to identify potential mechanisms of action and biological effects associated with these exposures. A future case-control study initially identifying exposure as proximity to the landfill should be extended with more advanced methods to measure exposures, for instance exposure questionnaires investigating occupational, environmental and dietary exposures, satellite data on air pollution, untargeted metabolomics of blood and tissue, and multiplexed immunohistochemistry of thyroid tissue to identify pollutants. These studies will enhance our knowledge on the potential additive, synergistic or antagonistic effects of various pollutants and will be of great importance to better understand the effects of environmental pollution on thyroid cancer.

## Figures and Tables

**Table 1:** Information on environmental risk factors associated with thyroid function/cancer from the Fresh Kills Landfill.

Exposure Pathway	Contaminants of Concern	Peak Concen-Tration (µg/m^3^)	Most Conservative CV (µg/m^3^)	Carcinogen?		EDC?	
				IARC ^a^	ECHA	EPA	ES
Air	Particulate matter:			1	Not listed	Not listed	Not listed
	* PM10	2289	50				
	* TSP	648	75				
	PM10-Cadmium	0.00467	0.0005	1	Carcinogenic/Suspected mutagenic	Not listed	EDC
	TSP-Cadmium	0.0516	0.0005				
Surface water	**≥1 surface water concentration exceeded the CV**:	**Mean highest concentration** *	**Drinking water CV (ppb)**				
	Cadmium	13.3	7	1	Carcinogenic/Suspected mutagenic	Not listed	EDC
	Lead	100	15	2B	May be carcinogenic	Not listed	Not listed
	Vanadium	126	30	2B	-	Not listed	Not listed
Sediment	**≥1 soil concentration exceeded the CV**:	**Mean highest concentration** *	**Soil CV (ppb)**				
	Cadmium	14.4	0.4	1	Carcinogenic/Suspected mutagenic	Not listed	EDC
	Lead	545	400	2B	May be carcinogenic	Not listed	Not listed
	Vanadium	63.9	6	2B	-	Not listed	Not listed
Food chain (fish, shellfish)	**Exceeded health-based regulatory limits**:	**Highest range of concentrations measured**					
	Cadmium	Eastern oyster: 2.920–8160 ppb		1	Carcinogenic/Suspected mutagenic	Not listed	EDC
	Lead	Horse mussel: 4409–6290 ppb		2B	May be carcinogenic	Not listed	Not listed
	Dioxins	Blue crab: 202.0–283.2 ppt		≥1 **	Not listed ^	Not listed	TCD
	PCBs	Blue crab: 4.40–13.90 ppm		1	Not listed ^	EDC	TCD
	DDT	American eel: 1700–6895 ppb		2A	Suspected carcinogenic	Not listed	EDC

* Mean value of the highest concentrations measured (including Main Creek, Richmond Creek, Fresh Kills, and Arthur Kill). ** Various agents containing compound listed. ^ No group classification provided. ^a^ IARC classification: Group 1: Carcinogenic to humans; Group 2A: Probably carcinogenic to humans; Group 2B: Possibly carcinogenic to humans; Group 3: Not classifiable as to its carcinogenicity to humans. CV: Comparison values; DDT: 4,4’-dichlorodiphenyltrichloroethane; EDC: endocrine disrupting chemical; PCB: polychlorinated biphenyl; PM: particulate matter; ppb: parts per billion; ppm: parts per million; ppt: parts per trillion; TDC: thyroid disrupting chemical; TSP: Total suspended particles.

## Data Availability

Publicly available datasets were analyzed in this study. This data can be found here: https://www.health.ny.gov/statistics/cancer/registry/nyspaced/faq.htm (accessed on 28 November 2021).

## Supplementary Materials

The following are available online at https://www.mdpi.com/article/10.3390/toxics9120325/s1, Table S1: Information on exposures from the Fresh Kills Landfill. Figure S1: Age-adjusted thyroid cancer incidence rates by sex for Staten Island (1995–2018). Figure S2: Annual percent change in age-adjusted thyroid cancer rates on Staten Island by sex between 1995 and 2018.
