# Supplementary material for: High Thyroid Cancer Incidence Rate in a Community near a Landfill: A Descriptive Epidemiological Assessment
Source: Toxics. 2021 Dec 1;9(12):325. doi: 10.3390/toxics9120325 (PMC8705650; doi:10.3390/toxics9120325)
Supplement: Supplementary file 1 [file toxics-09-00325-s001.zip › toxics-1456374-supplementary.pdf]

toxics

MDPI

Table S1. Information on potential exposures from the Fresh Kills Landfill [1].

Exposure pathway	Source of contamination	Route of exposure	Time of measure-ment	Contaminants of concern	Peak concen-tration (μg/m³)	Most conservative CV (μg/m³)	Carcinogen? IARC[2 a]	ECHA[3]	EPA[4]	EDC? ES[5,6]
Air	Volatilization of contaminants, emissions, and dust	Inhalation	1990–1999	1,1,2-Trichloroethane	1.73	0.01	3	Suspected	+	Not listed
				1,2-Dichloroethane	0.94	0.01	2B	Carcinogenic	+	Not listed
				Particulate matter:<br>* PM10<br>* TSP	2,289<br>648	50<br>75	1	Not listed	Not listed	Not listed
				Acetaldehyde	9.93	0.27	2B	Carcinogenic/ Suspected mutagenic	+	Not listed
				Acrolein	1.021	0.009	2A	-	Not listed	Not listed
				Benzene	5.53	0.03	1	Carcinogenic/ Mutagenic	+	Not listed
				TSP-Beryllium	0.0013	0.0004	1	Carcinogenic	Not listed	Not listed
				n-Butyraldehyde	0.47	NA*	Not listed	-	Not listed	Not listed
				PM10-Cadmium<br>TSP-Cadmium	0.00467<br>0.0516	0.0005<br>0.0005	1	Carcinogenic/ Suspected mutagenic	Not listed	EDC
				Carbon Tetrachloride	0.51	0.01	2B	Suspected carcinogenic	+	Not listed
				Chloroform	2.1	0.008	2B	Suspected carcinogenic	Not listed	Not listed
				PM10-Chromium<br>TSP-Chromium	0.0367<br>0.0479	0.00002<br>0.00002	3	-	Not listed	Not listed
				Formaldehyde	13.32	0.05	1	Carcinogenic/ Suspected mutagenic	Not listed	Not listed
				Hexanal	0.838	NA*	Not listed	-	Not listed	Not listed
				Methacrolein	0.51	NA*	Not listed	-	Not listed	Not listed

				Methylene Chloride	6.36	0.85	2A	Suspected carcinogenic	Not listed	Not listed
				PM10-Nickel<br>TSP-Nickel	0.2223<br>0.222	0.002<br>0.002	2B	Suspected carcinogenic	Not listed	Not listed
				Propionaldehyde	16.866	0.04	3	-	Not listed	Not listed
				PM10-Sulfate	37.8	NA*	≥2A**	?***	Not listed	Not listed
				Tetrachloroethylene	65.23	0.17	2A	Suspected carcinogenic	+	Not listed
				m-Tolualdehyde	0.19	NA*	Not listed	-	Not listed	Not listed
				Trichloroethylene	2.6	0.08	1	Carcinogenic/ Suspected mutagenic	+	Not listed
				Vinyl Chloride	0.42	0.01	1	Carcinogenic	Not listed	Not listed
**Ground-water**	Leaching of contaminants	Ingestion<br>Dermal contact	1991-1996	**>50% of the samples exceeded the CV**:	**% of samples over CV**	**Highest reported concentration (ppb)**				
				Arsenic<br>- Shallow wells<br>-Intermediate wells	 81.6<br>55.8	 52,800<br>130°	1	-^	Not listed	EDC
				Barium<br>-Shallow wells	 76.8	 12,800°	Not listed	-	Not listed	Not listed
				Benzene<br>-Shallow wells	 59.2	 58	1	Carcinogenic/ Mutagenic	+	Not listed
				Boron<br>-Shallow wells<br>-Intermediate wells<br>-Deep wells	 98.1<br>66.8<br>80.4	 10,700<br>10,500<br>2,920	Not listed	-	Not listed	Not listed
				Manganese<br>-Shallow wells<br>-Intermediate wells<br>-Deep wells	 81.6<br>94.9<br>78.9	 52,800<br>40,300<br>21,800°	Not listed	-	Not listed	Not listed
				Total Ammonia<br>-Shallow wells	 95.1		Not listed	-	Not listed	Not listed

						6,010,000 °				
				**≥ 1 surface water concentration exceeded the CV:**	**Drinking water CV (ppb)**	**Mean highest concentration°°**				
**Surface water**	Leaching of contaminants	Ingestion Dermal contact	1991-1994	Ammonia	3,000	4,553	Not listed	-	Not listed	Not listed
				Antimony	3	30	≥2B**	-	Not listed	Not listed
				Arsenic	0.02	500	1	-^	Not listed	EDC
				Beryllium	0.01	5	1	Carcinogenic	Not listed	Not listed
				Boron	100	2,433	Not listed	-	Not listed	Not listed
				Cadmium	7	13.3	1	Carcinogenic/ Suspected mutagenic	Not listed	EDC
				Lead	15	100	2B	May be carcinogenic	Not listed	Not listed
				Manganese	50	354.3	Not listed	-	Not listed	Not listed
				Selenium	20	750	3	-	Not listed	Not listed
				Silver	50	70	Not listed	-	Not listed	Not listed
				Sulfate	500,000	2,367,500	≥2A**	?***	Not listed	Not listed
				Thallium	0.4	413.3	Not listed	-	Not listed	Not listed
				Vanadium	30	126				
				**≥ 1 soil concentration exceeded the CV:**	**Soil CV (ppb)**	**Mean highest concentration°°**				
**Sediment**	Leaching of contaminants	Ingestion Dermal contact	1991-1994	Antimony	0.8	13.8	≥2B**	-	Not listed	Not listed
				Arsenic	0.5	51.1	1	-^	Not listed	EDC
				Barium	100	1,625	Not listed	-	Not listed	Not listed
				Boron	20	28.5	Not listed	-	Not listed	Not listed
				Cadmium	0.4	14.4	1	Carcinogenic/ Suspected mutagenic	Not listed	EDC
				Chromium	6	251	3	-	Not listed	Not listed
				Lead	400	545	2B	May be carcinogenic	Not listed	Not listed
				Manganese	300	485	Not listed	-	Not listed	Not listed

				Nickel	40	64	2B	Suspected carcinogenic	Not listed	Not listed
				Vanadium	6	63.9	2B	-	Not listed	Not listed
				Zinc	600	712	Not listed	-	Not listed	Not listed
**Food chain (fish, shellfish)**	Contamination of surface water and sediment	Ingestion	1995-1997	**Exceeded health-based regulatory limits**:	**Highest range of concentrations measured**					
				Cadmium	Eastern oyster: 2.920-8,160 ppb		1	Carcinogenic/ Suspected mutagenic	Not listed	EDC
				Lead	Horse mussel: 4,409-6,290 ppb		2B	May be carcinogenic	Not listed	Not listed
				Dioxins	Blue crab: 202.0-283.2 ppt		≥1**	?***	Not listed	TCD
				Furans	Blue crab: 155.7-253.0 ppt		≥1**	?***	Not listed	Not listed
				PCBs	Blue crab: 4.40-13.90 ppm		1	?***	+	TCD
				DDT	American eel: 1,700-6,895 ppb		2A	Suspected carcinogenic	Not listed	EDC
				Chlordane	White perch: 195-380 ppb		2B	Suspected carcinogenic	Not listed	EDC

*no published health-based comparison value available. **Various agents containing compound listed. *** No group classification provided. ^ Arsenic acid is classified as carcinogenic. ^^ Toluene dilsocyanate listed. ° Value considered to be an outlier. °° Mean value of the highest concentrations measured (including Main Creek, Richmond Creek, Fresh Kills, Arthur Kill). [a] IARC classification: Group 1: Carcinogenic to humans; Group 2A: Probably carcinogenic to humans; Group 2B: Possibly carcinogenic to humans; Group 3: Not classifiable as to its carcinogenicity to humans. CV: Comparison values; DDT: 4,4'-dichlorodiphenyltrichloroethane; EDC: endocrine disrupting chemical; PCB: polychlorinated biphenyl; PM: particulate matter; ppb: parts per billion; ppm: parts per million; ppt: parts per trillion; TDC: thyroid disrupting chemical; TSP: Total suspended particles.

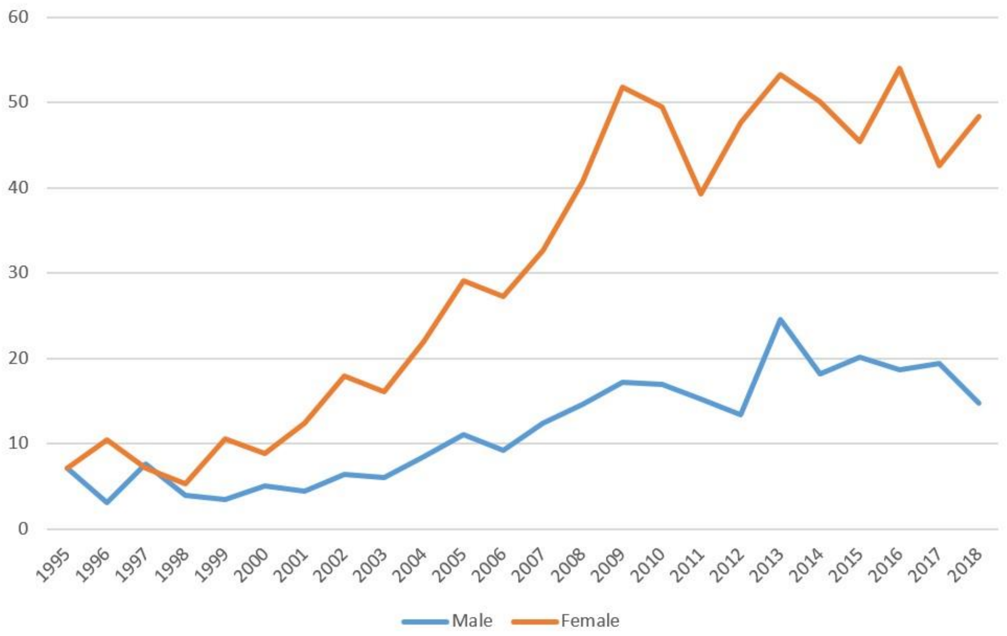

Figure S1. Age-adjusted thyroid cancer incidence rates by sex for Staten Island (1995–2018).

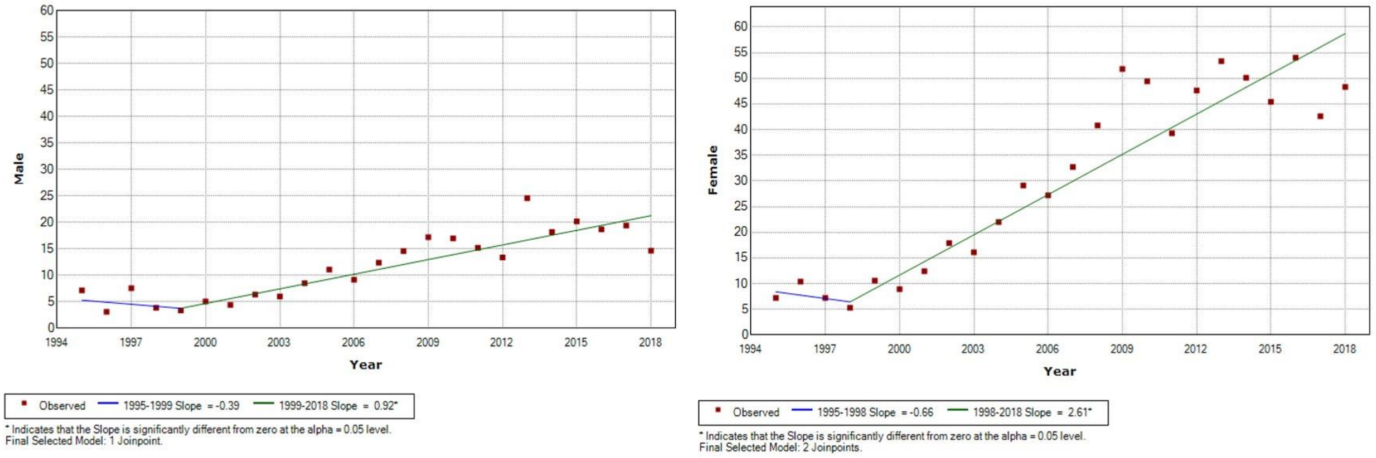

Figure S2. Annual percent change in age-adjusted thyroid cancer rates on Staten Island by sex between 1995 and 2018.

References:

1. Agency for Toxic Substances and Disease Registry (ATSDR). 1998. Health Consultation Fresh Kills Landfill, Staten Island, Richmond County, New York CERCLIS No. NYD980506943.
2. International Agency for Research on Cancer – IARC Monographs on the identification of carcinogenic hazards to humans https://monographs.iarc.who.int/list-of-classifications. Accessed on 21 September 2021.
3. European Chemicals Agency (ECHA)- Information on Chemicals. https://echa.europa.eu/information-on-chemicals. Accessed on 21 September 2021.
4. United States Environmental Protection Agency (EPA) Endocrine Disruptor Screening Program - Second list of chemicals for tier 1 screening under the Safe Drinking Water Act (SDWA) https://www.epa.gov/endocrine-disruption/overview-second-list-chemicals-tier-1-screening-under-endocrine-disruptor. Accessed on 21 September 2021.
5. Diamanti-Kandarakis, E., Bourguignon, J.P., Giudice, L.C., et al. Endocrine-disrupting chemicals: an Endocrine Society scientific statement. *Endocr Rev.* **2009** Jun;30(4):293-342.
6. Gore, A.C., Chappell, V.A., Fenton, S.E., et al. EDC-2: The Endocrine Society's Second Scientific Statement on Endocrine-Disrupting Chemicals. *Endocr Rev.* **2015** Dec;36(6):E1-E150.
